# Complete Mitochondrial Genome Characterization of *Schrankia costaestrigalis* (Insecta: Erebidae: Hypenodinae) and Its Phylogenetic Implication

**DOI:** 10.3390/genes14101867

**Published:** 2023-09-26

**Authors:** Xuyuan Gao, Yu Bai, Xiaodong Jiang, Xiuzhen Long, Dewei Wei, Zhan He, Xianru Zeng, Yonghao Yu

**Affiliations:** 1 Key Laboratory of Green Prevention and Control on Fruits and Vegetables in South China Ministry of Agriculture and Rural Affairs/Guangxi Key Laboratory of Biology for Crop Diseases and Insect Pests/Plant Protection Research Institute, Guangxi Academy of Agricultural Sciences, Nanning 530007, China; gxy@gxaas.net (X.G.); nkyjxd@163.com (X.J.); lxz@gxaas.net (X.L.); wdw@gxaas.net (D.W.); happy-hezhan@163.com (Z.H.); 2 College of Mathematics & Information Science, Guiyang University, Guiyang 550005, China; baiyu403@163.com

**Keywords:** *Schrankia costaestrigalis*, Hypenodinae, mitochondrial genome, phylogenetic analysis

## Abstract

The pinion-streaked snout *Schrankia costaestrigalis* is a new potato pest that has recently been recorded in China. In this study, we analyzed the complete mitochondrial genome of *S. costaestrigalis*. The results revealed the mitogenome (GenBank: OQ181231) to occur as a circular DNA molecule of 16,376 bp with 51.001% AT content, including 13 protein-coding genes (PCGs), 22 transfer RNA (tRNA) genes, 2 ribosomal RNA (rRNA) genes, and 1 control region. Notably, the PCGs exhibited typical ATN (Met) start codons, including *cox1*, which deviated from the usual CGA start codon observed in other lepidopteran mitogenomes, and followed the conventional TAN stop codons. The 22 tRNA genes demonstrated the ability to form a cloverleaf structure, with the exception of trnS1-NCU, which lacked the DHU arm present in other Erebidae mitogenomes. Additionally, conserved motifs like “ATAGA + poly-T (19 bp) stretch” and five microsatellite-like elements (TA) were identified in the AT-rich region. The phylogenetic trees revealed that the Hypenodinae subfamily forms an independent lineage closely related to Erebinae and Catocalinae. The comprehensive mitogenome of S. costaestrigalis will greatly enhance future studies focused on the molecular classification and phylogenetic understanding of the Hypenodinae subfamily within the larger family Erebidae.

## 1. Introduction

**Figure 1:**
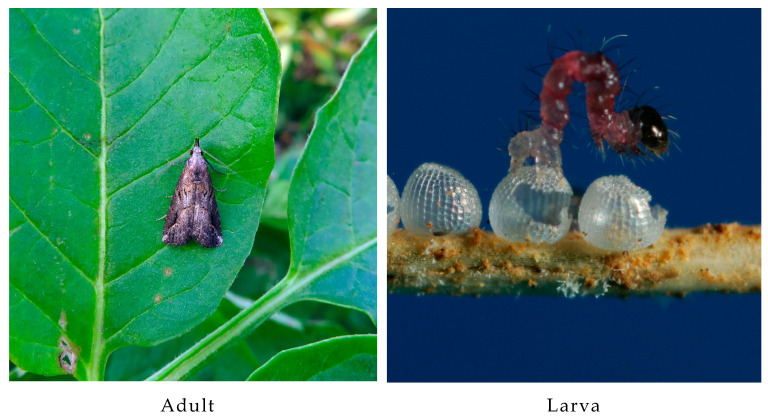

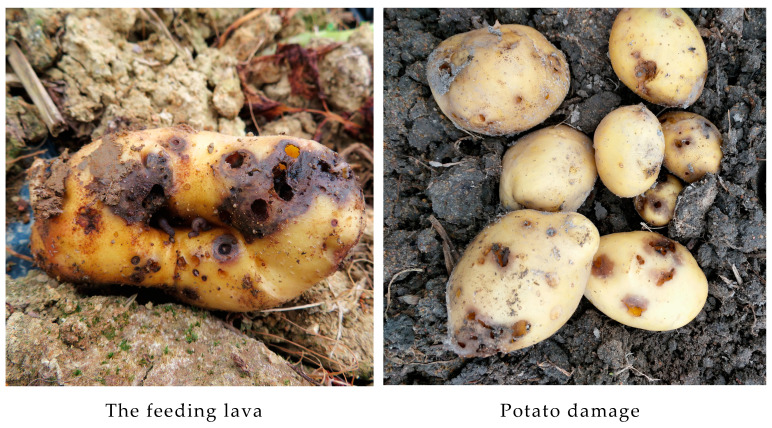
The images of *S. costaestrigales* and its damage.

The pinion-streaked snout *Schrankia costaestrigalis* (Stephens, 1834), a species of Lepidoptera in the family Erebidae, subfamily Hypenodinae, belongs to the genus *Schrankia* Hübner (1825) (Figure 1). It is distributed in Europe, Central and North Asia, North Africa, and Australia [1]. This pest was previously defined as a general insect that feeds on various herbaceous and woody plants [2]. However, the pest broke out in the potato’s main producing area of Yulin City, Guangxi, China in February 2017, affecting an area of 287.34 ha. This was the first report of *S. costaestrigalis* in China [3]. The only other report of this pest-damaging crop was on the broad bean *Vicia faba* in Japan [4]. The damage rate to potato tubers generally ranged between 50% and 80%, with values reaching 100% in some fields. This resulted in a yield loss of 5 million kg and caused a significant impact on the winter potato industry in Guangxi. Furthermore, there has been a rise in potato production in China since 2015, making it the fourth largest crop in the country after rice, wheat, and corn [5]. Thus, the occurrence of this pest poses a potential threat to the commercial production of potatoes in China and food security in general.

S. *costaestrigalis* is not typically regarded as an agricultural pest, and research on the pest is lacking [3,4]. In particular, the pest’s origin and development in China remain unclear. Mitochondria, which are cellular organelles, play a crucial role in cellular energy metabolism and respiration. Studying the mitochondrial genome of a rare agricultural pest can help us to better understand its population genetic structure and genetic diversity, and thus allow us to infer its origin and distribution. Moreover, by comparing the mitochondrial genome of this pest with those of other related species, scientists can also clarify its classification and evolutionary relationships, providing a scientific basis for pest control [6]. The interrelationships between major subfamilies and tribes within the Erebidae family were analyzed utilizing molecular data from nuclear and mitochondrial genes. Clade Hypenodinae was lifted to a subfamily status, which made the genera *Schrankia* Hübner and *Luceria* Walker be associated more closely [7]. And by comparing the mitochondrial genome of *Orthaga olivacea* Warre with that of other lepidopteran insects, it was confirmed that *O. Olivacea* belongs to the Pyralidae family, which provides a reference for the pest’s control [8].

Insect mitochondrial genomes, known as mitogenomes, are circular, double-stranded DNA molecules that typically range in length from 15 to 19 kb. They comprise a total of 37 genes, encompassing various types including 13 protein-coding genes (PCGs), 2 ribosomal RNA genes (rRNAs), 22 transfer RNA genes (tRNAs), and a control region (CR) [9]. Despite the usefulness of next-generation sequencing (NGS) in mitogenome assembly and phylogenetic analysis of insects [9], it can be challenging to bridge the gap between contigs, particularly in regions with high A+T content. To address this issue, PCR amplification and Sanger sequencing can be employed to successfully fill in the gaps in the mitogenome sequence.

This research focuses on the sequencing and characterization of the mitogenome of *S. costaestrigalis*, representing the first complete mitogenome within the Hypenodinae subfamily. By utilizing a combination of next-generation sequencing (NGS) and Sanger sequencing techniques, we successfully obtained the full sequence. Additionally, we constructed phylogenetic trees using 24 mitogenomes, including 22 mitogenomes of the Erebidae family and 2 outgroup mitogenomes. This analysis enhances our understanding of the phylogenetic placement of *S. costaestrigalis* within the broader context of the Erebidae family. The findings of this study are valuable for reconstructing the phylogenetic relationships among species within the Erebidae family.

## 2. Materials and Methods

### 2.1. Animal Materials and DNA Extraction

The individuals of *S. costaestrigalis* were collected from Renhou Village (22.639° N, 110.056° E), Renhou Town, Yuzhou District, Yulin City, Guangxi Zhuang Autonomous Region, China, on 20 March 2022. They were continuously raised in the lab. To extract genomic DNA (gDNA) from the pupae, the Qiagen DNeasy Blood and Tissue Extraction kit (Qiagen, Germantown, MD, USA) were employed. The purified gDNA was detected using a NanoPhotometer^®^ spectrophotometer (Implen, Los Angeles, CA, USA), while the concentration was determined using a Qubit^®^ 2.0 fluorometer (Life Technologies, Carlsbad, CA, USA).

### 2.2. Illumina and Sanger Sequencing

Sequencing libraries for the quality-checked gDNA were generated using a TrueLib DNA Library Rapid Prep Kit for Illumina sequencing (Illumina, Inc., San Diego, CA, USA). The libraries were subjected to size distribution analysis using an Agilent 2100 bioanalyzer (Agilent Technologies, Inc., Santa Clara, CA, USA), followed by real-time PCR quantitative testing. The successfully generated libraries were sequenced using an Illumina NovaSeq 6000 platform (Illumina, Inc., San Diego, CA, USA). A total of 150-bp paired-end reads with a 300-bp insert library were generated. Three pairs of primers designed to match generally conserved regions of the published target mtDNA were used to amplify short fragments from *nad3*-*nad5*, rrnL and the control region (CR) (Supplementary Table S1). The PCR products were cloned into pMD18-T vectors (Takara, Kyoto, Japan) and subsequently sequenced, or they were sequenced directly by the dideoxy nucleotide procedure, using an ABI 3730 automatic sequencer (Applied Biosystems, Foster City, CA, USA) [10] (Supplementary File S1).

### 2.3. Raw Reads Cleaning and Mitogenome Assembly

The high-quality clean reads were filtered from the raw data. This was achieved by employing the fastp tool (version 0.23.2) [11], which is a widely used software for read quality filtering (source code available at: https://github.com/OpenGene/fastp, accessed on 9 January 2023). The quality control (QC) criteria applied to ensure the reliability of the raw reads were as stated:

(1) Trimming adapter sequences longer than six bases;
(2) Removal of reads with >0 unidentified nucleotides (N);
(3) Removal of reads with >20% bases with Phred quality < Q20;
(4) Removal of reads with <15 bases.

The cleaned data were assembled into the mitogenome of *S. costaestrigalis* using the following two mitochondrial assembly processes:

(1) NOVOPlasty v4.3.1 [12], with default parameters and the *S*. *costaestrigalis* isolate Scos02 *cox1* gene (GenBank: EF061755.1) [13] as the initial template.
(2) The mitogenome of *S*. *costaestrigalis* was constructed from high-quality cleaned reads using the de novo assembly software GetOrganelle v1.7.6.1 [14]. Default parameters were employed, and the *cox1* gene from the *S. costaestrigalis* isolate Scos02 (GenBank: EF061755.1) [13] served as the initial reference sequence for assembly.

### 2.4. Annotation and Analysis of the Mitochondrial Genome

To investigate the bias in nucleotide composition, the AT-skew [(A − T)/(A + T)] and GC-skew [(G − C)/(G + C)] of the sequences were estimated using a formula proposed by Perna and Kocher [15]. The *S. costaestrigalis* mitogenome was initially annotated using GeSeq version 2.03, an online tool available at https://chlorobox.mpimp-golm.mpg.de/geseq.html (accessed on 9 January 2023) [16]. For tRNA identification, the tRNAscan-SE v2.0.7 [17], ARWEN v1.2.3 [18], and BLAT v36×7 [19] were utilized, with the *Eudocima phalonia* mitogenome (NC_032382.1) as a reference. Manual correction of start and stop codons of the protein-coding genes (PCGs) was performed, referencing the mitogenomes of Eudocima phalonia. The gene order and orientation were established and displayed by utilizing CGView (https://proksee.ca/, accessed on 9 January 2023) [20]. The relative synonymous codon usage (RSCU) values for the 13 PCGs were calculated using MEGA v11.0.13 [21]. Analysis and visualization of tRNA secondary structures were performed using forna (http://rna.tbi.univie.ac.at/forna/, accessed on 9 January 2023) [22,23].

### 2.5. Phylogenetic Inference

In this study, we performed a phylogenetic analysis using the mitogenomic sequences of 22 species belonging to the Erebidae family. Two additional species were used as outgroup references. (Table 1). *Lepisma saccharina* and *Corydidarum magnifica* were selected as the outgroups. Nucleotide sequences of 13 PCGs from mitogenoms were used to construct the phylogenetic relationships within *S*. *costaestrigalis* utilizing PhyloSuite version 1.2.2 [24], including MAFFT [25], ModelFinder [26,27], and MrBayes version 3.2.7 [28]. Alignment was performed using MAFFT version 7 with the default settings. The nucleotide matrix was used for the phylogenetic analysis with two methods: Bayesian inference (BI) in MrBayes 3.2.7 and the maximum likelihood (ML) method with MEGA v11.0.13 [21]. The best-fit edge-unlinked partition model for BI was selected using PhyloSuite version 1.2.2 [24] with the Bayesian Information Criterion (BIC), employing ModelFinder [26,27] (Supplementary Table S2). A BI analysis was conducted for each matrix (two parallel runs, 20,000,000 generations). The initial 25% of the generated trees were discarded as burn-in, and the average standard deviation of split frequencies for the remaining trees was 0.002572 (<0.01), indicating convergence. The ML tree was constructed using MEGA v11.0.13 [21], and the best-fit model, as determined by BIC scores, was the General Time Reversible (GTR) model coupled with a discrete γ distribution (+G) consisting of 5 rate categories. For the ML analysis, 1000 bootstrap resampling replicates were performed to assess node support values. The generated phylogenetic trees were visualized using the Interactive Tree Of Life (iTOL) tool (source: https://itol.embl.de/) (accessed on 9 January 2023) [29].

## 3. Results and Discussion

### 3.1. Sequencing, QC, Mitogenome Organization and Base Composition of S. costaestrigalis

From a 300 bp insert library, a total of approximately 5.64 Gb raw reads were generated. Subsequently, the fastp software [9] was employed to obtain approximately 5.37 Gb of high-quality clean reads. The Q20 (percentage of bases with quality value ≥ 20), Q30 (percentage of bases with quality value ≥ 30), and G+C content values of the clean reads were 97.82%, 92.84%, and 34.84%, respectively (Table 2).

Using NOVOPlasty, the high-quality cleaned short reads, which accounted for 0.20% of the total reads from the mitogenome, allowed for the near-complete assembly of the *S. costaestrigalis* (OQ181231.1) mitogenome. This assembly achieved 100% coverage of the mitogenome with a high average-read depth of 675 times. By reducing repetitive sequences, the assembly process was able to generate a comprehensive and accurate representation of the *S. costaestrigalis* mitogenome [12]. This contains the mitochondrial sequence results from four possible combinations, ranging in length from 16,094 to 16,101 bp. Although the circular mitochondrial genome sequence was successfully assembled using GetOrganelle [14], the length of the sequence was 15,550 bp, and filling the gap in CR with high A+T content between contigs proved to be difficult. We sequenced the three regions using sanger sequencing, including *nad3*-*nad5*, rrnL, and CR, and manually assembled a complete mitogenome which consists of traditional circular DNA molecules. The mitogenome exhibited the longest length of 16,376 bp in the family Erebidae (Table 1). The mitogenome of *S*. *costaestrigalis* contains 39.57% A, 41.68% T, 7.32% G, and 11.43% C, showing an obvious AT bias with a 81.25% A+T content, which was slightly lower compared to *Dysgonia stuposa* [38]. Specifically, the major strand of the *S. costaestrigalis* mitogenome exhibited an AT-skew of −0.026 and a GC-skew of −0.219. These values indicated a compositional bias on the major strand, with a slight excess of T nucleotides over A nucleotides, and a strong excess of C nucleotides over G nucleotides. The AT and GC bias were similar to other mitogenomes, in the family Erebidae, such as *Dysgonia stuposa* [38] and *Hydrillodes repugnalis* [40].

**Figure 2:**
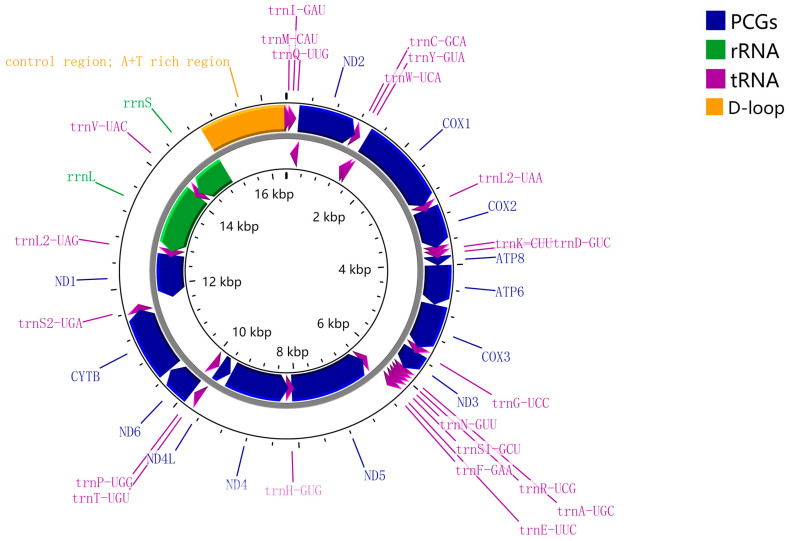
Mitogenome pattern map of *S*. *costaestrigalis*. Arrows indicated the orientation of gene transcription.

The *S*. *costaestrigalis* mitogenome comprised 13 PCGs, 1 CR, and 22 tRNA genes, and 2 rRNA genes (Figure 1). The arrangement and orientation of genes in the *S. costaestrigalis* mitogenome were consistent with other mitogenomes in the family Erebidae. On the majority strand (J-strand), 23 genes, including 9 PCGs and 14 tRNAs, were encoded. On the minority strand (N-strand), there were four PCGs, eight tRNAs, and two rRNAs (Figure 2 and Table 3). Within these genes, there were 12 instances of overlap, totaling 37 bp, with the longest overlapping region found between trnW-UCA and trnC-GCA (Table 3). Additionally, there were 17 intergenic spacer regions, spanning a total of 113 bp, with the longest spacer occurring between trnS1-GCU and trnE-UUC (Table 3).

### 3.2. Protein-Coding Genes

On the majority strand of the *S. costaestrigalis* mitochondrial genome, nine protein-coding genes (*cob*, *cox1*, *cox2*, *cox3*, *atp6*, *atp8*, *nad2*, *nad3*, and *nad6*) were encoded, while four genes (*nad1*, *nad4*, *nad4l*, and *nad5*) were encoded on the minority strand (refer to Figure 2 and Table 3). For all 13 protein-coding genes, the start codon followed the traditional ATN (Met) pattern, as indicated in Table 3. Specifically, only *nad6* began with an ATC start codon, *cox2* and *nad5* began with an ATA start codon, *cox1*, *atp6*, *cox3*, *nad4, nad4l*, and cob had an ATG start codon, and *nad2*, *atp8*, *nad3*, and *nad1* began with an ATT start codon. It was notable that the start codon for *cox1*, commonly observed in lepidopteran mitogenomes as CGA, was ATG in the *S. costaestrigalis* mitogenome (in contrast to previous findings [40]). Moreover, all 13 protein-coding genes exhibited a conventional TAA stop codon. Specifically, ten genes (*nad2*, *cox1*, *atp8*, *atp6*, *cox3*, *nad3*, *nad4l*, *nad6*, *cob*, and *nad1*) terminated with a TAA stop codon, while three genes (*cox2*, *nad5*, and *nad4*) had an incomplete stop codon (T), which could be presumably modified to TAA through post-transcriptional polyadenylation.

**Figure 3:**
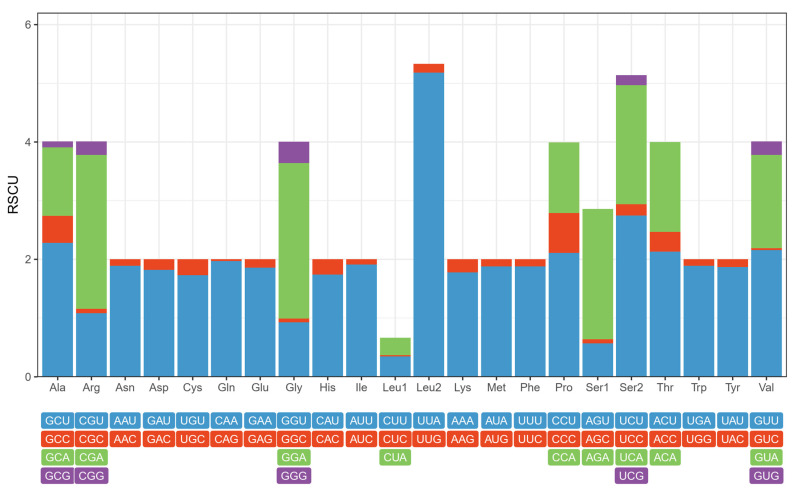
Relative synonymous codon usage (RSCU) of *S*. *costaestrigalis* mitogenome.

The analysis of relative synonymous codon usage (RSCU) was performed on the 13 protein-coding genes, which consisted of a total of 3711 codons excluding the start and stop codons. The RSCU analysis unveiled the presence of codon usage bias within the *S. costaestrigalis* mitogenome (refer to Figure 3 and Supplementary Table S3). Furthermore, when examining the frequency of amino acids (as shown in Supplementary Table S3), it was observed that Leu was the most abundant (558 occurrences), followed by Ile (455) and Phe (365). Looking specifically at the codon usage counts, it was found that UUA (482) was the predominant codon for Leu, AUU (435) followed Ile, and UUU (343) followed Phe. While most codons were present in the *S. costaestrigalis* mitogenome, there were a few exceptions. Specifically, the codons CUG for Leu, CCG for Pro, and AGG for Ser1 were absent, indicating a lack of GC-rich synonymous codons with G at the third codon position [38]. Furthermore, the thirteen protein-coding genes displayed a preference for A and T nucleotides, as outlined in Supplementary Table S4.

### 3.3. Transfer and Ribosomal RNA Genes

**Figure 4:**
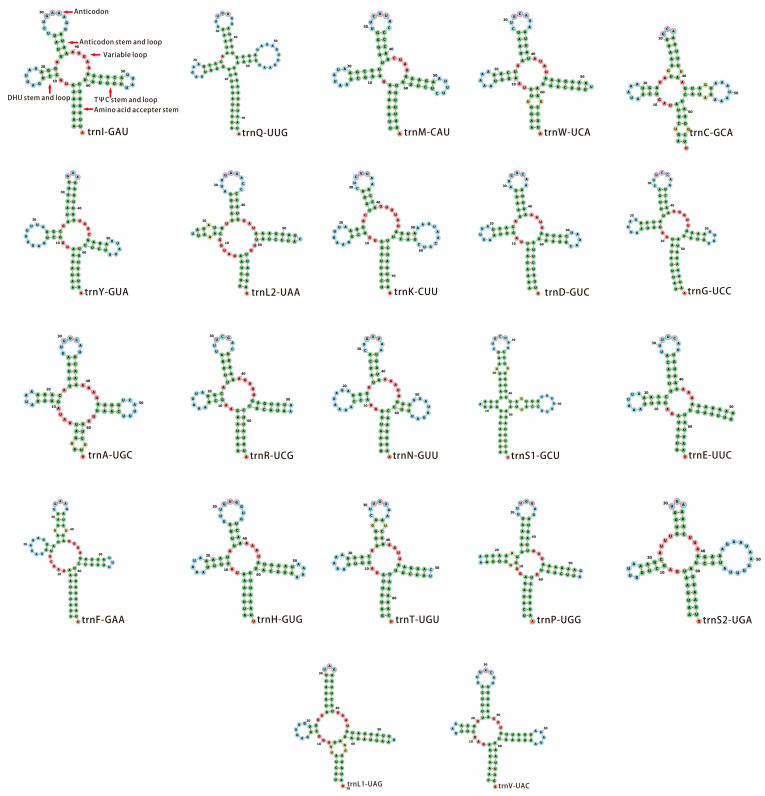
Secondary structure of 22 tRNAs from the *S*. *costaestrigalis* mitogenome. Mismatched base pairs were visually highlighted in yellow, while matched base pairs were shown in green. Bases within loops were represented in blue, while the nucleotide outlines of the anticodon were indicated in red.

**Figure 5:**
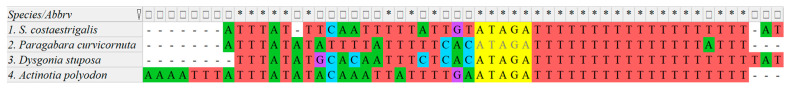
Alignment of initiation site for the control region of four species mitogenomes. The Yellow indicates the conserved motif ATAGA.

The 22 tRNA genes in the mitochondrial genome of *S. costaestrigalis* were distributed among the protein-coding genes (PCGs). Among these tRNAs, 14 were encoded on the majority strand, while 8 were encoded on the minority strand (Figure 2 and Table 3). Their lengths varied between 64 bp (trnT-UGU) and 73 bp (trnQ-UUG and trnK-CUU) (Table 3 and Supplementary Table S4), and they all possessed the characteristic cloverleaf secondary structure (depicted in Figure 4). The secondary structures of most tRNAs closely reflected similarity with the one observed in *Dysgonia stuposa* [38] and *Hydrillodes repugnalis* [40] mitogenomes. However, trnS1-GCU in the *S. costaestrigalis* mitogenome exhibited a different structure, featuring a dihydrouridine (DHU) stem (Figure 4) of 3 bp. The diversity in tRNA secondary structures reflected the evolutionary variations among species. The length of the anticodon tRNA stems ranged from 4 bp (trnI-GAU, trnK-CUU, trnH-GUG, and trnS2-UGA) to 9 bp (trnS1-GCU) (Figure 4). The DHU stem length varied from 2 bp (trnF-GAA and trnL1-UAG) to 6 bp (trnP-UGG) (Figure 5), with most falling between 3 and 4 bp. The TΨC stem length varied between 3 bp (trnN-GUU) to 8 bp (trnL1-UAG) (Figure 4), with the majority being between 4 and 5 bp. Two types of mismatched base pairs were identified in tRNAs, namely A-G base pairs and non-canonical G-U base pairs (Figure 5). The DHU stem of trnP-UGG and the TΨC stem of trnC-GCA contained A-G base pairs (Figure 5). The anticodon stems of trnC-GCA, trnS1-GCU, trnF-GAA, and trnT-UGU; the amino acid acceptor stems of trnW-UCA, trnC-GCA, trnA-UGC, and trnL1-UAG; the DHU stem of trnL2-UAA; and G-U base pairs were present in the TΨC stem of trnS1-GCU (Figure 5).

The rrnL and rrnS were 1400 and 781 bp long, respectively, and were encoded on the N strand. The rrnL was located between trnL1 and trnV, while rrnS resided between trnV and CR. This is similar to that of the *Dysgonia stuposa* and *Hydrillodes repugnalis* mitogenomes [38,40]. The AU-skews of the rrnL and rrnS were 0.062 and 0.063, respectively, and corresponding GC-skews were determined as 0.406 and 0.345, respectively (Supplementary Table S4). The rrnL and rrnS showed a markedly high A+T content (83.64 and 85.53%, respectively) bias (Supplementary Table S4), similar to that of the *Laelia suffusa* mitogenome [44].

### 3.4. Control Region

The CR, also denoted as the AT-rich region or D-loop, had a length of 1421 base pairs and exhibited a high AT content of 91.77%. It was situated between the rrnS and trnM-CAU genes (Figure 2 and Supplementary Table S3). The AT content of the CR in the mitogenome of *S. costaestrigalis* was marginally greater compared to that of *Leucoma salicis* [45]. Analysis of the CR revealed compositional biases on the majority strand. Specifically, the AT-skew was calculated to be −0.051, indicating a slight excess of T nucleotides over A nucleotides. Similarly, the GC-skew was determined to be −0.197, indicating a significant excess of C nucleotides over G nucleotides. These findings suggest a notable compositional bias in favor of certain nucleotides on the major strand of the CR. The conserved structures that connected the motif “ATAGA + poly-T (19 bp) stretch” [31,36,44,45,49] were located right flanking of the rrnS gene within the CR (Figure 5 and Supplementary File S2). TR1, TR3, TR6, TR8, and TR10 were varied and typical microsatellite-like elements (TA) (Supplementary Table S5).

### 3.5. The Construction of Phylogenetic Trees

**Figure 6:**
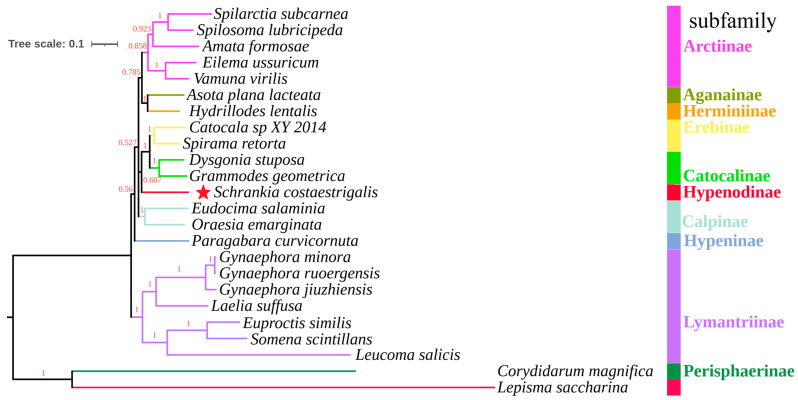
The phylogenetic tree was inferred from the nucleotide sequences of 13 mitogenome PCGs using BI methods. Red-colored node indicators represent the posterior probability values on the Bayesian inference (BI) phylogenetic tree. Red star indicates the newly determined *S*. *costaestrigalis*. *Lepisma saccharina* and *Corydidarum magnifica* are outgroups.

**Figure 7:**
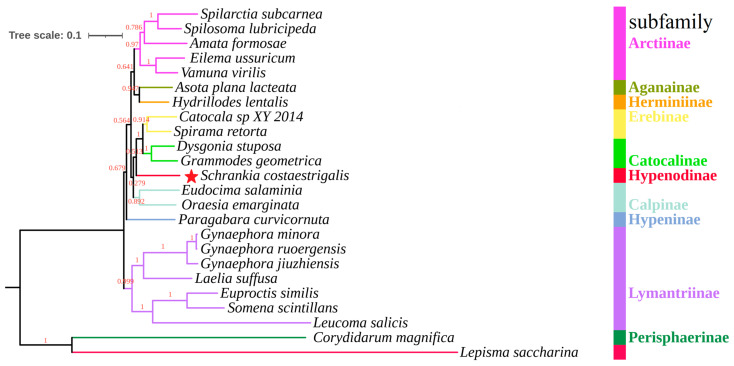
The phylogenetic tree was inferred from the nucleotide sequences of 13 mitogenome PCGs using ML methods. The nodes on the tree are represented with red-colored ML bootstrap support values. Red star indicates the newly determined *S*. *costaestrigalis*, *Lepisma saccharina* and *Corydidarum magnifica* as outgroups.

The phylogenetic trees were constructed using nucleotide sequences of the 13 PCGs from 24 mitogenomes (as depicted in Figure 6 and Figure 7). Although the BI tree demonstrated considerably higher support values compared to the ML tree in the same dataset, the trees exhibited significantly low support values in the same branches. In the two trees, nine subfamilies were clustered as ((((Arctiinae + (Herminiinae + Aganainae)) + ((Erebinae + Catocalinae) + Hypenodinae) + Calpinae) + Hypeninae) + Lymantriinae) (Figure 6 and Figure 7), which mirrored earlier phylogenetic investigations [50]. At the subfamily level, the subfamily Hypenodinae is an independent lineage and is closely related to the subfamilies Erebinae and Catocalinae based on the topology of the trees (Figure 6 and Figure 7). As is known, the species of the subfamily Hypenodinae are small-sized moths and distributed only in the world [51]. They were placed as a tribe before, but had a kinship with the subfamily Erebinae and Catocalinae [7]. These results will contribute to establishing a molecular framework for the classification and phylogeny within the Erebidae family, specifically focusing on the Hypenodinae subfamily.

## 4. Conclusions

The pinion-streaked snout *S*. *costaestrigalis* is distributed in Europe, Central and North Asia, North Africa, and Australia [1], yet information on its mitogenome molecular phylogenetic is lacking. Therefore, in the current study, we assembled the *S*. *costaestrigalis* mitogenome. This was the first complete mitogenome in the subfamily Hypenodinae and is observed to have similar structural characteristics and nucleotide composition, compared to other previously reported mitogenomes of the family Erebidae. We identified 13 PCGs, 1 CR, and 22 tRNA genes, and 2 rRNA genes in our assembled mitogenome. All of the 13 protein-coding genes were initiated with a common start codon, typically ATN (Met), including *cox1*, which was initiated with the CGA start codon in most of the lepidopteran mitogenomes. These genes were terminated using the standard TAN stop codons. The 22 tRNAs exhibit a characteristic cloverleaf structure, typical for mitochondrial genomes. Notably, trnS1-NCU lacks the DHU arm, distinguishing it from other mitogenomes within the family Erebidae. Additionally, the CR presents a conserved motif “ATAGA + poly-T (19 bp) stretch”. Phylogenetic analysis has revealed that the subfamily Hypenodinae forms an independent lineage, closely related to the subfamilies Erebinae and Catocalinae. Given the diverse nature of the Erebidae family and the current limitations in mitogenome data, a more comprehensive understanding of the phylogeny within the family Erebidae would necessitate the inclusion of additional mitogenomes.

## Figures and Tables

**Table 1:** The sequences of 24 mitogenomes employed for the construction of phylogenetic trees.

Family	Subfamily	Species	Whole Length	GenBank	Reference
Erebidae	Aganainae	*Asota plana lacteata*	15,416 bp	KJ173908.1	[30]
	Arctiinae	*Amata formosae*	15,463 bp	KC513737.1	[31]
		*Eilema ussiricum*	15,344 bp	MN696172.1	[32]
		*Spilarctia subcarnea*	15,441 bp	KT258909.1	[33]
		*Spilosoma lubricipedum*	15,375 bp	MT591568.1	[34]
		*Vamuna virilis*	15,417 bp	KJ364659.1	[30]
	Calpinae	*Eudocima salaminia*	15,597 bp	MW683337.1	[35]
		*Oraesia emarginata*	16,668 bp	MW648382.1	[36]
	Catocalinae	*Grammodes geometrica*	15,728 bp	KY888135.1	[37]
		*Dysgonia stuposa*	15,721 bp	MK262707.1	[38]
	Erebinae	*Catocala deuteronympha*	15,671 bp	KJ432280.1	[30]
		*Spirama retorta*	15,652 bp	MT013356.1	[39]
	Herminiinae	*Hydrillodes repugnalis*	15,570 bp	MH013484.1	[40]
	Hypeninae	*Paragabara curvicornuta*	15,532 bp	KT362742.1	[41]
	Hypenodinae	*Schrankia costaestrigalis*	16,376 bp	OQ181231.1	This study
	Lymantriinae	*Euproctis similis*	15,437 bp	KT258910.1	[42]
		*Gynaephora jiuzhiensis*	15,859 bp	KY688085.1	[43]
		*Gynaephora minora*	15,801 bp	KY688086.1	[43]
		*Gynaephora rouergensis*	15,803 bp	KY688083.1	[43]
		*Laelia suffusa*	15,502 bp	MN908152.1	[44]
		*Leucoma salicis*	15,334 bp	MT230535.1	[45]
		*Somena scintillans*	15,410 bp	MH051839.1	[46]
Lepismatidae		*Lepisma saccharina*	15,244 bp	MT108230.1	[47]
Blaberidae	Perisphaerinae	*Corydidarum magnifica*	16,627 bp	MW630139.1	[48]

**Table 2:** Summary of sequencing reads for *S*. *costaestrigalis*.

Raw Reads Base (bp)	Raw Reads Num	Q20 (%)	Q30 (%)	Clean Reads Base (bp)	Clean Reads Num	Q20 (%)	Q30 (%)	G + C (%)
5,639,351,400	37,595,676	96.91	91.53	5,369,244,032	36,000,780	97.82	92.84	34.84

**Table 3:** The mitogenome organization of *S*. *costaestrigalis*.

Gene	Strand	Location	Size (bp)	Anticodon	Start Codon	Stop Codon	Intergenic Nucleotides
trnM	J	1–68	68	CAU			
trnI	J	71–138	68	GAU			2
trnQ	N	134–206	73	UUG			−5
*nad2*	J	210–1220	1011		ATT	TAA	3
trnW	J	1222–1288	67	UCA			1
trnC	N	1281–1346	66	GCA			−8
trnY	N	1349–1415	67	GUA			2
*cox1*	J	1418–2959	1542		ATG	TAA	2
trnL2	J	2954–3022	69	UAA			−6
*cox2*	J	3022–3703	682		ATA	T	−1
trnK	J	3706–3778	73	CUU			2
trnD	J	3778–3846	69	GUC			−1
*atp8*	J	3846–4007	162		ATT	TAA	−1
*atp6*	J	4001–4678	678		ATG	TAA	−7
*cox3*	J	4678–5466	789		ATG	TAA	−1
trnG	J	5468–5535	68	UCC			1
*nad3*	J	5538–5888	351		ATT	TAA	2
trnA	J	5902–5966	65	UGC			13
trnR	J	5968–6033	66	UCG			1
trnN	J	6035–6102	68	GUU			1
trnS1	J	6106–6173	68	GCU			3
trnE	J	6200–6264	65	UUC			26
trnF	N	6262–6330	69	GAA			−3
*nad5*	N	6329–8072	1744		ATA	T	0
trnH	N	8073–8140	68	GUG			0
*nad4*	N	8141–9479	1339		ATG	T	0
*nad4l*	N	9495–9782	288		ATG	TAA	16
trnT	J	9787–9850	64	UGU			4
trnP	N	9850–9917	68	UGG			−1
*nad6*	J	9919–10,446	528		ATC	TAA	1
*cob*	J	10,462–11,613	1152		ATG	TAA	15
trnS2	J	11,612–11,678	67	UGA			−2
*nad1*	N	11,700–12,638	939		ATT	TAA	21
trnL1	N	12,638–12,707	70	UAG			−1
rrnL	N	12,708–14,107	1400				0
trnV	N	14,108–14,174	67	UAC			0
rrnS	N	14,175–14,955	790				0
CR	J	14,956–16,376	1421				0

Abbreviations: J, J-strand (the majority strand); N, N-strand (the minority strand).

## Data Availability

The deposition details for the DNA sequences are as follows: The raw data is available at NCBI’s Sequence Read Archive under the accession numbers SRR21850432. The associated BioProject and Bio-Sample numbers are PRJNA888939 and SAMN31222830, respectively.

## Supplementary Materials

The following supporting information can be downloaded at: https://www.mdpi.com/article/10.3390/genes14101867/s1, Table S1: Primers of *nad3–nad5*, rrnL and the control region of the *S*. *costaestrigalis* mitogenome; Table S2: The best-fit edge-unlinked partition model of the nucleotide sequences of 13 PCGs of 24 mitogenomes for BI; Table S3: T Codon number and RSCU of the PCGs; Table S4: Composition and skewness of genes and the control region; Table S5: Tandem repeats of the control region; File S1: Raw data of the Sanger sequencing; File S2: Alignment of the control region of four species mitogenomes.
